# Role of the *MtLUX*-*MtRVE1* regulatory module in auxin-mediated root development and nodule formation in *Medicago truncatula*

**DOI:** 10.1093/hr/uhag124

**Published:** 2026-04-06

**Authors:** Tao Fan, Li-ping Wang, Jing Li, Ming-kang Yang, Yue Chen, Meng-jiao Jin, Liang Chen, Liang-fa Ge, Wei Huang

**Affiliations:** Guangdong Basic Research Center of Excellence for Precise Breeding of Future Crops, Guangdong Provincial Key Laboratory of Protein Function and Regulation in Agricultural Organisms, State Key Laboratory for Conservation and Utilization of Subtropical Agro-Bioresources, College of Life Sciences, South China Agricultural University, Guangzhou, Guangdong 510642, China; Guangdong Basic Research Center of Excellence for Precise Breeding of Future Crops, Guangdong Provincial Key Laboratory of Protein Function and Regulation in Agricultural Organisms, State Key Laboratory for Conservation and Utilization of Subtropical Agro-Bioresources, College of Life Sciences, South China Agricultural University, Guangzhou, Guangdong 510642, China; Guangdong Basic Research Center of Excellence for Precise Breeding of Future Crops, Guangdong Provincial Key Laboratory of Protein Function and Regulation in Agricultural Organisms, State Key Laboratory for Conservation and Utilization of Subtropical Agro-Bioresources, College of Life Sciences, South China Agricultural University, Guangzhou, Guangdong 510642, China; Guangdong Basic Research Center of Excellence for Precise Breeding of Future Crops, Guangdong Provincial Key Laboratory of Protein Function and Regulation in Agricultural Organisms, State Key Laboratory for Conservation and Utilization of Subtropical Agro-Bioresources, College of Life Sciences, South China Agricultural University, Guangzhou, Guangdong 510642, China; Guangdong Basic Research Center of Excellence for Precise Breeding of Future Crops, Guangdong Provincial Key Laboratory of Protein Function and Regulation in Agricultural Organisms, State Key Laboratory for Conservation and Utilization of Subtropical Agro-Bioresources, College of Life Sciences, South China Agricultural University, Guangzhou, Guangdong 510642, China; Guangdong Basic Research Center of Excellence for Precise Breeding of Future Crops, Guangdong Provincial Key Laboratory of Protein Function and Regulation in Agricultural Organisms, State Key Laboratory for Conservation and Utilization of Subtropical Agro-Bioresources, College of Life Sciences, South China Agricultural University, Guangzhou, Guangdong 510642, China; Guangdong Basic Research Center of Excellence for Precise Breeding of Future Crops, Guangdong Provincial Key Laboratory of Protein Function and Regulation in Agricultural Organisms, State Key Laboratory for Conservation and Utilization of Subtropical Agro-Bioresources, College of Life Sciences, South China Agricultural University, Guangzhou, Guangdong 510642, China; Guangdong Subcenter of the National Center for Soybean Improvement, College of Agriculture, South China Agricultural University, Guangzhou 510642, China; Guangdong Basic Research Center of Excellence for Precise Breeding of Future Crops, Guangdong Provincial Key Laboratory of Protein Function and Regulation in Agricultural Organisms, State Key Laboratory for Conservation and Utilization of Subtropical Agro-Bioresources, College of Life Sciences, South China Agricultural University, Guangzhou, Guangdong 510642, China

## Abstract

The circadian clock synchronizes a multitude of biological events with environmental changes, thereby optimizing plant growth and development. In legumes, nodule formation, a pivotal process that sustains symbiotic nitrogen fixation, is one such event regulated by the circadian clock. Nevertheless, the mechanisms underlying the circadian clock’s regulation of nodule formation and nitrogen fixation are still poorly elucidated. Herein, we unveil that the core clock gene *LUX ARRHYTHMO* (*LUX*) exerts a crucial role in modulating nodule formation and root development via auxin biosynthesis pathways in the model legume *Medicago truncatula*. Our findings indicate that MtLUX directly associates with the promoter of *MtRVE1*, a clock output gene involved in auxin biosynthesis, both *in vivo* and *in vitro*, thereby repressing its expression. Biochemical and genetic data further corroborate that the *MtLUX-MtRVE1* regulatory module adjusts root architecture and nodule formation through the fine-tuning of auxin biosynthesis. These discoveries reveal a mechanism whereby the circadian clock integrates hormonal pathways to regulate nodule formation, thereby linking circadian regulation, auxin biosynthesis, and nitrogen fixation in legumes. This research lays the groundwork for enhancing legume growth and nitrogen acquisition under fluctuating environmental conditions.

## Introduction

Nitrogen fixation is the biological process by which atmospheric nitrogen (N₂) is converted into ammonia (NH₃), which is then usable by plants. Legumes, in symbiosis with rhizobia, fix atmospheric N₂, enriching soil fertility and reducing the need for chemical fertilizers, thus lessening agriculture’s environmental footprint. As global populations rise and arable land decreases, enhancing legumes’ nitrogen-fixing ability is crucial for ensuring food security and addressing climate change [1, 2]. Therefore, the research into legume nodulation and nitrogen fixation is invaluable for advancing both basic science and sustainable agricultural practices.

Nitrogen-fixing rhizobia engage in symbiotic interactions with legumes in specialized organs known as root nodules. Within these nodules, rhizobia reside as intracellular symbionts, converting atmospheric nitrogen into ammonia, thereby alleviating the nitrogen deficiency in the host plant [3, 4]. In return, the host plant supplies the rhizobia with organic compounds such as dicarboxylic acids, which the bacteria utilize for their growth and metabolism, establishing a mutually beneficial relationship [5]. The intricate relationship between legumes and rhizobia not only facilitates nodule formation but also ensures the plant’s ability to utilize the nitrogen-fixing capacity of the rhizobia, enhancing plant growth and productivity. The development of root nodules in legumes is a sophisticated process governed by intricate genetic regulation. Nodule formation is a meticulously orchestrated process that initiates with the recruitment of rhizobia by plant root exudates, proceeds through signal recognition, immune modulation, infection thread development, nodule differentiation, nitrogen fixation, and culminates in nitrogen assimilation and transport, achieving a mutually beneficial symbiosis between legumes and rhizobia [6–9]. Consequently, the investigation of biological nitrogen fixation is of paramount significance in terms of enhancing soil quality via plant-mediated biological nitrogen fixation, augmenting crop productivity, and fostering the advancement of ecological agriculture.

During the symbiosis of *Lotus japonicus* with rhizobial bacteria, plant hormones, notably cytokinin, significantly influence the development of root nodules and the cyclic modulation of the host’s transcriptomic pattern [10, 11]. In addition, key regulatory genes, including those involved in root architecture such as *SHORTROOT* (*SHR*) and *SCARECROW* (*SCR*), are instrumental in the early stages of nodule initiation [12, 13]. The *SHR*–*SCR* module in legumes, such as *Medicago truncatula*, may operate upstream in the signaling cascade, orchestrating nodule formation by local auxin biosynthesis and cortical cell divisions [12]. This module is pivotal in establishing the auxin gradients necessary for nodule initiation and development through the precise control of PIN-FORMED (PIN) protein polar localization within cortical cells, thereby facilitating the symbiotic relationship with nitrogen-fixing rhizobia [14, 15]. Additionally, recent studies have proved that auxin affects root nodule formation, with species-specific mechanisms across legumes. In soybean, GmPIN-dependent polar auxin transport is also involved in nodule development, with *GmPIN1* overexpression suppressing nodule primordia initiation [16]. Auxin response factors (ARFs) are transcription factors that regulate growth and development mediated by auxin. In soybean, *GmARF8* overexpression reduces the number of root nodules [17]. Conversely, in *M. truncatula*, *MtARFs* positively regulate root system development and nitrogen-fixing nodule formation [18]. Further studies reveal that the formation of these nodules is determined by a local maximum concentration of the phytohormone auxin at the site of the primordium, similar to the development of other plant organs [19, 20]. The dynamic transport of auxin, particularly the polar auxin transport (PAT) mediated by PIN proteins, plays a pivotal role in modulating various aspects of soybean nodule development [14]. This process is suggested to be a prerequisite for the proper interaction between legumes and rhizobia, as indicated by studies demonstrating the importance of establishing an auxin gradient. Moreover, the transcription factor *NODULE INCEPTION 2 (NIN2*) regulates soybean nodule zonation and cell differentiation by modulating auxin levels via *GRETCHEN HAGEN 3* (*GH3*) auxin-amido synthetases. NIN2 activation reduces auxin, promoting differentiation, while elevated auxin enhances NIN2 accumulation, forming a feedback loop [21]. This highlights the pivotal role of auxin homeostasis in nodule development. However, the precise role of auxin in nodule development remains not fully elucidated. The circadian clock is an inner timing mechanism that can anticipate the recurring environmental fluctuations caused by the rotation of the Earth around its axis, and coordinate biological processes to synchronize with the approximately 24-h cycle of day and night [22, 23]. In plants, as well as across eukaryotic species, numerous transcriptional feedback loops are central to clock function. *LUX ARRHYTHMO* (*LUX*), also referred to as *PHYTOCLOCK1* (*PCL*), encodes a GARP transcription factor that acts as a nighttime repressor of circadian gene expression at the core of the central oscillator [24, 25]. *LUX* is one of the few oscillator genes whose mutants cause arrhythmic disruptions, and it is associated with numerous TTFLs. The evening complex (EC), composed of EARLY FLOWERING 3 (ELF3), EARLY FLOWERING 4 (ELF4), and LUX, binds to the promoters of nearly all oscillator and numerous output genes [26]. It plays a central role in maintaining clock rhythmicity and regulating various biological processes, including growth, development, and stress responses. Whether the circadian clock regulates nitrogen fixation and nodule formation in leguminous plants has been a topic of significant interest to scientists for decades [27, 28]. Emerging studies reveal that the core oscillator gene *LHY* in *M. truncatula* is a central regulator of nodulation, providing insights into the integration of circadian control with nitrogen assimilation and plant development. Mutations in the *MtLHY* gene lead to altered circadian rhythmicity, which is associated with a compromised nodulation phenotype in legumes, potentially due to the disruption of timely gene expression crucial for the successful establishment of symbiosis with rhizobia [29]. In *M. truncatula*, the rapid damping of gene expression rhythms for *LHY*, *TOC1*, *PRR5/9*, *PRR7*, *GI*, and *CCR2* in *mtlux* mutants impairs nodule formation and function, highlighting the importance of these genes in the establishment of the symbiotic relationship with rhizobia [30]. In soybean, alterations in the *GmTOC1* gene affect nodule formation, with loss of function leading to increased nodules and overexpression inhibiting nodulation, indicating that *GmTOC1* plays a regulatory role in balancing the nodule development to meet the plant’s nitrogen requirements [31]. Importantly, the successful establishment and maintenance of the symbiotic relationship between legumes and rhizobia may depend on the circadian regulation of key genes. Specifically, the rhythmic expression of genes like *PHEHYLALANINE AMMONIA LYASE* (*PAL*), which is crucial for flavonoid synthesis and the initial signaling to recruit rhizobia, is orchestrated by the plant’s internal clock [27]. Additionally, flavonoid-*O*-methyltransferases (OMTs) are used to investigate the response of rhizobium to various flavonoids. In *M. truncatula*, *ChOMT1* and *OMT2* play important role in nod gene activation and promote nodulation [32]. Furthermore, genes involved in nodule organogenesis such as *NODULE INCEPTION* (*NIN)* and genes associated with nitrogen fixation, including those encoding for nitrogenase components and *NOD FACTOR PERCEPTION* (*MtNFP*), are also under circadian control [33, 34]. This precise temporal regulation ensures that the various stages of nodule formation, from bacterial infection to nitrogen assimilation, are synchronized with the plant’s daily metabolic cycles, highlighting the integral role of the circadian clock in the complex dynamics of legume-rhizobia symbiosis.

The circadian clock is a pivotal regulator of growth and developmental processes in the above-ground parts of plants, but its influence on root morphology has received limited attention. Additionally, the intricate mechanisms underlying the circadian clock influences nodulation, and nitrogen fixation in the roots of leguminous plants are not well understood. Here, we show that the overexpression of the plant circadian clock’s core gene, *MtLUX*, leads to a root phenotype with increased auxin levels and enhanced nodulation. In contrast, the mutants exhibit a phenotype indicative of reduced auxin and diminished nodulation. Using the auxin reporter transgenic line *DR5::VENUS*, it was verified that *LUX* regulates auxin synthesis. The upregulation of auxin biosynthetic genes *YUCs* in the mutants suggests the indirect activation of *YUCs* by *MtLUX*. The previous studies have characterized LUX as a repressor. RNA-seq analysis of the roots from *mtlux* mutants and complemented seedlings has uncovered ‘regulation of hormone levels’ among the top 20 enriched pathways. Focusing on genes involved in hormone regulation, we identified co-expression modules significantly associated with auxin distribution. Within the hormone biosynthetic process (GO:0042446) and auxin biosynthetic process (GO:0009851), *YUC5* and *YUC10* emerged as central nodes based on network topology. Both genes were downregulated in *mtlux* mutant roots, consistent with their roles in auxin biosynthesis. In addition, *RVE1*, a circadian clock output gene implicated in auxin regulation, was differentially expressed. These results suggest that circadian regulation may influence auxin distribution through transcriptional modulation of *YUC* genes, thereby contributing to root system architecture. Subsequently, our biochemical and genetic studies have validated that *MtLUX* contributes to auxin biosynthesis by inhibiting the expression of *MtRVE1*, which in turn affects root system development and the formation of nodules.

## Results

### The *MtLUX* gene regulates root development and nodule formation via auxin signaling

The majority of studies have focused on the regulation of aerial parts by the clock, while fewer have examined its control of root morphology and rhythmicity [35]. *LUX* is a core oscillator gene of the plant circadian clock. Given the species- and tissue-specific characteristics of the circadian clock, we investigated the rhythmic expression patterns of *MtLUX* in roots by generating transgenic *M. truncatula* harboring the *MtLUX* promoter fused to the luciferase (LUC) reporter gene (*MtLUXpromoter::LUC*). Utilizing this transgenic line, we analyzed the rhythmic expression profiles of *MtLUX* in both shoot and root tissues. Our findings reveal that *MtLUX* exhibits rhythmic variations in all tissues examined, with a significantly longer expression period in roots compared to shoots (Fig. S1). This contrasts with observations in *Arabidopsis thaliana* and aligns with our previous findings [36]. In comparison with other oscillator genes, robust oscillations of *MtLUX* were observed in both shoots and roots, suggesting a pivotal regulatory role of *MtLUX* in the morphology and functionality of the roots.

**Figure 1:**
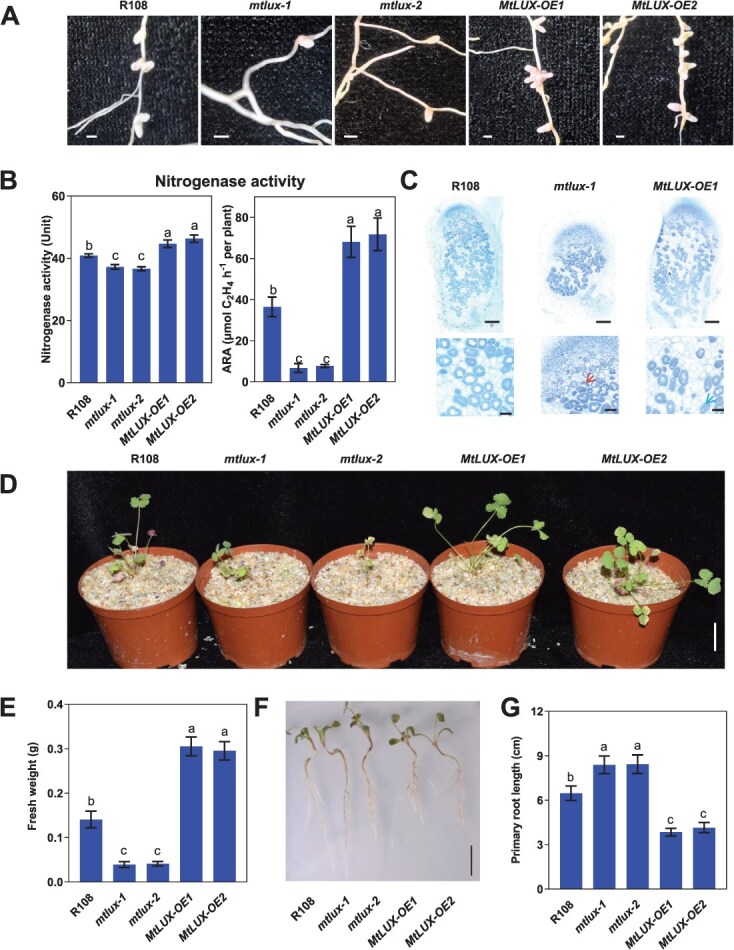
A role for *MtLUX* in the auxin-related phenotypes of *mtlux* mutants and *MtLUX* overexpressing (OE) lines. (A) Nodule growth after 21 days of rhizobial inoculation. Scale bars: 1 mm. (B) Assessment of rhizobial activity in root nodules at 21 days postinoculation. The left panel shows nitrogenase activity per nodule measured using the Plant Nitrogenase (NITS) ELISA kit. The right panel shows nitrogenase activity per plant determined by the ARA. At least 12 independent plants were analyzed. Statistical significance was determined by Student’s *t* test. Different letters indicate significant differences (*P* < 0.05). (C) Toluidine blue-stained longitudinal semithin sections of nodules from the *mtlux* mutant and overexpression lines. Sections were prepared from resin-embedded samples and observed under light microscopy. The bottom panel shows higher-magnification views of the regions indicated in the top panel. Red arrows indicate bacteria that may be undergoing degradation. Blue arrows indicate putative bacteroids. Scale bars: 200 μm (top) and 50 μm (bottom). (D) Above-ground phenotypes of transformed lines 21 days postinoculation. Scale bars: 2 cm. (E) Analysis of fresh weight in above-ground plant parts was conducted 21 days postinoculation. Samples were collected from at least eight plant lines, and statistical analysis was performed using *t* test. Different letters ‘a–c’ indicate statistically significant differences (*P* < 0.001). (F) Root phenotypes of 10-day-old *MtLUX* mutants and overexpressing lines. Scale bars: 1 cm. (G) Statistical analysis of primary root length. Different letters ‘a–c’ indicate significant differences in descending order. Each experiment included at least six biological replicates. Each experiment was conducted in triplicate.

Therefore, we conducted phenotypic analysis of root morphology and nodulation in the *mtlux* mutant and transgenic overexpression plants, revealing significant differences when compared to the wild type, as reported in a previous study [30]. Consequently, we conducted a phenotypic analysis of nodulation in transgenic overexpressing plants *MtLUX-OE1* and *MtLUX-OE2*, as well as in *MtLUX* CRISPR/Cas9-edited mutants *mtlux-1* and *mtlux-2*. Compared to the wild type, the *MtLUX-OE1* and *MtLUX-OE2* exhibited significantly more nodules, while the nodule count in the *mtlux-1* and *mtlux-2* was substantially lower (Fig. 1A). Furthermore, nitrogenase activity assays revealed key differences relative to the R108 line, *mtlux* mutant nodules exhibited significantly reduced activity, while *MtLUX*-overexpressing lines (*MtLUX-OE*) showed enhanced activity (Fig. 1B). Nitrogenase activity per nodule in R108, measured using the Plant Nitrogenase (NITS) ELISA kit, was higher than that in the *mtlux* mutant but lower than in *MtLUX-OE* lines (Fig. 1B, left). Consistently, per-plant nitrogenase activity assessed by the acetylene reduction assay (ARA) showed the same trend (Fig. 1B, right). These results indicate that *MtLUX* positively modulates nitrogenase activity at both the nodule and whole-plant levels. Further morphological analysis of toluidine blue-stained nodule sections revealed that the *mtlux* mutant displayed irregular cellular organization compared with the R108 control (Fig. 1C). Additionally, following inoculation with rhizobia, the *MtLUX-OE* lines showed markedly enhanced growth in nitrogen-free nutrient solution relative to both the wild type and the mutants (Fig. 1D). Under low-nitrogen conditions, the *MtLUX-OE* exhibited significantly elevated shoot biomass compared to controls, while the *mtlux* mutants displayed lower shoot biomass (Fig. 1E). These findings highlight an important regulatory role of *MtLUX* in controlling nodule formation.

Specifically, the primary root length of the *MtLUX* CRISPR/Cas9-edited mutants *mtlux-1* and *mtlux-2* was considerably longer than that of the wild type, whereas the primary root length of the *MtLUX* overexpression lines *MtLUX-OE1* and *MtLUX-OE2* was shorter (Fig. 1F and G). When grown on 1/2 MS medium containing 2 μM of the auxin transport inhibitor *N*-1-naphthylphthalamic acid (NPA), both mutant (*mtlux-1* and *mtlux-2*) and overexpression lines (*MtLUX-OE1* and *MtLUX-OE2*) showed primary root lengths similar to the wild-type R108 (Fig. S2). This suggests that the *mtlux* mutants exhibit an auxin-deficient phenotype, while the *MtLUX* overexpression lines display an auxin accumulation phenotype. Moreover, we observed venus fluorescence intensity in the primary root tips of *DR5::VENUS/R108* transgenic lines and hybrid seedlings derived from *DR5::VENUS/R108* and *mtlux-1* lines. The results showed lower hormone concentration in *mtlux-1*. These results suggest that *MtLUX* may regulate root development through the auxin biosynthesis pathway (Fig. S3).

**Figure 2:**
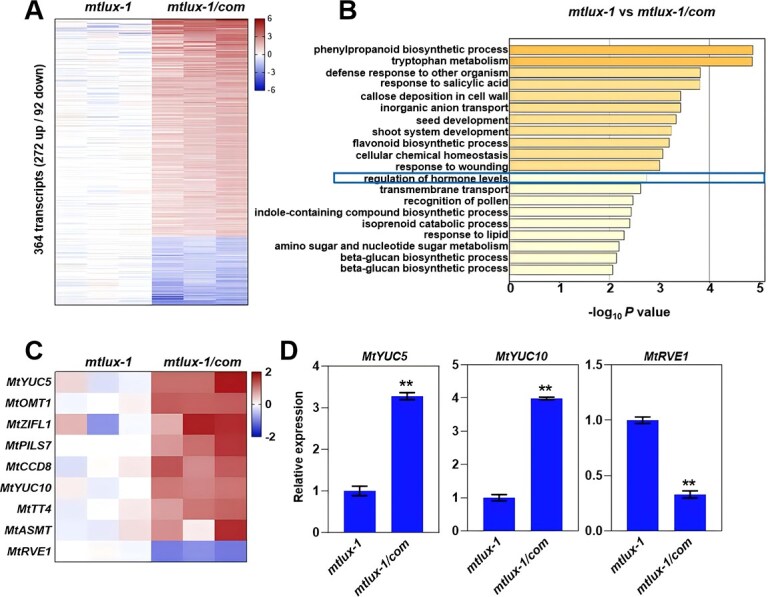
Root RNA-seq data of *mtlux-complementary* and *mtlux mutant* plants. Roots from 4-week-old plants were collected for RNA sequencing. (A) DEGs from RNA-seq data. (B) Enrichment analyses of Gene Ontology (GO) biological processes, KEGG pathways and the WikiPathways. Bar graph depicting enriched biological process ontologies across input gene lists, colored by *P* values. The top 20 enriched gene ontology biological processes are presented here. (C) Heat map illustrating genes involved in the hormone biosynthetic process in root DEGs. (D) The expression analysis of growth hormone synthesis genes *MtYUC5*, *MtYUC10*, and *MtRVE1* in *mtlux* mutants and complementary lines. Roots of 10-day-old seedlings were sampled at ZT6h. The relative transcript levels were normalized to the abundance of the reference gene *MtACTIN* and are presented as means ± SD of three biological replicates (*n* = 3). Asterisks indicate significant difference (^**^P < 0.05). Each experiment was conducted in triplicate.

Recent studies have demonstrated auxin’s role in root development and nodule formation. However, how *MtLUX* modulates these processes via auxin regulation remains unclear. To investigate how *MtLUX* influences auxin synthesis, we performed RNA-seq analysis of root tissue to identify related genes (Fig. 2). Our RNA-seq analysis of root tissue from the *mtlux-1* mutant and the complementation line *MtLUX promoter*::*LUX*/*mtlux-1* identified 364 differentially expressed genes (DEGs), including 272 upregulated genes and 92 downregulated genes (Fig. 2A, Table S2). The DEGs play pivotal roles in multiple biological processes, including plant growth and development, hormone signaling pathways, and stress response mechanisms. Furthermore, enrichment analysis of Gene Ontology (GO) biological processes, KEGG pathways, and the WikiPathways (an online collaborative pathway resource, including biological pathways) identified significant associations (Fig. 2B). The significant enrichment of phenylpropanoid biosynthesis and cell wall callose deposition indicates that *MtLUX* is involved in cell wall formation, hormone metabolism, and stress response, suggesting a significant role of *MtLUX* in nodule formation. In addition, ‘regulation of hormone levels’, ‘hormone biosynthetic process’ and ‘auxin biosynthetic process’ within the ‘tryptophan metabolism’ pathway were among the top 20 enriched pathways, further suggesting that *MtLUX* may be involved in auxin biosynthesis (Fig. 2C, Table S3). Notably, the *YUC* family genes *YUC5* and *YUC10* emerged as central hubs in these pathways and were significantly downregulated in *mtlux* mutants compared with complementation lines (Fig. 2C). This reduced expression is consistent with their established roles in promoting auxin accumulation in roots. The *MtLUX* gene in plants typically functions as a transcriptional repressor. In the *mtlux* mutant, auxin biosynthesis genes (*YUC5* and *YUC8*) were downregulated, leading us to hypothesize that *MtLUX* indirectly regulates these genes. The observed downregulation of the auxin biosynthesis genes *MtYUC5* and *MtYUC10* in the mutant further supports this hypothesis. Subsequently, we performed quantitative polymerase chain reaction (qPCR) to analyze the expression of the downregulated *MtYUC5* and *MtYUC10* and the upregulated *MtRVE1,* all of which are involved in the auxin biosynthesis pathway. We confirmed that in the mutant roots, the expression levels of *MtYUC5*, *MtYUC10*, and *MtRVE1* were significantly reduced, correlating with the observed root phenotypes (Fig. 2D).

### MtLUX directly binds to the promoter of *MtRVE1* and represses its expression

LUX protein typically functions as repressor by binding to specific LUX binding sites (LBS) to regulate plant rhythmicity, growth, and development [37]. Among these downregulation of the auxin biosynthesis genes identified in RNA-seq, only the *MtRVE1* gene, which regulates auxin levels, contains an LBS site. In *Arabidopsis*, *RVE1* is a member of the Myb family linking the circadian clock to auxin biosynthesis. Therefore, *MtLUX* may regulate the expression of auxin biosynthesis genes by directly repressing *MtRVE1*, thereby influencing root growth and nodule development through the modulation of auxin levels.

**Figure 3:**
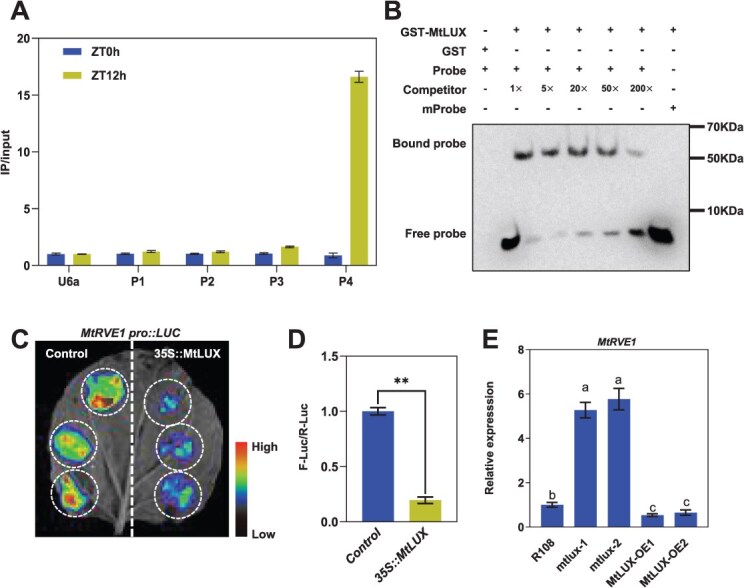
MtLUX binds to the promoter of *MtRVE1* and regulates its expression. (A) ChIP-qPCR analyses were conducted to assess MtLUX binding to the *MtRVE1* promoter. The upper figure indicates the structure of *MtRVE1* promoter, including LBS. Symbols labeled above the lines represent LBS in the 5′ → 3′ direction, while symbols labeled below the lines represent LBS in the 3′ → 5′ direction. Leaves from 4-week-old *MtLUXpro::MYC-MtLUX-GFP-2HA/mtlux-1* plants were collected at ZT0h and ZT12h for ChIP analysis. Fragment U6a was used as a negative control. The data were normalized to U6a at ZT0, which was set to 1. (B) EMSA demonstrated that MtLUX binds to the *MtRVE1* promoter. The orange-framed LBS in panel A located in subfragment P4 was chosen as the MtLUX binding site. The symbols ‘+’ and ‘−’ indicate presence or absence, respectively, of Probe, mutant probe (mProbe). (C and D) Dual-luciferase transient expression assay demonstrated MtLUX activation of the *MtRVE1* promoter. The MCS (multiple cloning site) served as the empty vector control, while MtLUX was driven by a full-length cauliflower mosaic virus (CaMV) 35S promoter in the effector constructs. Firefly luciferase was driven by the *MtRVE1* promoter in the reporter construct. *Renilla* luciferase (Rluc), driven by the 35S promoter in the reporter construct, was used as an internal control. This transient transcriptional expression analysis demonstrated MtLUX activation in the epidermal cells of *N. benthamiana* leaves. (D) The ratio of firefly luciferase (Fluc) to Renillaluciferase (Rluc). Data are presented as the mean ± SEM from at least four biological replicates. The statistical analysis was performed using the *t* test; asterisks indicate significant differences (^**^P < 0.05). The data were normalized by control (control = 1). (E) qPCR analysis of *MtRVE1* expression in R108 and *MtLUX* mutants at ZT12h. The relative transcript levels were normalized to the abundance of the reference gene *MtACTIN* and are presented as means ± SD of three biological replicates (*n* = 3). Different letters ‘a–c’ indicate significant differences in descending order. Each experiment was conducted in triplicate.

To verify whether MtLUX binds to the promoter of *MtRVE1 in vivo*, we utilized transgenic plants of *MtLUXpro::MYC-MtLUX-GFP-2HA/mtlux-1* and conducted chromatin immunoprecipitation (ChIP)-qPCR experiments. We identified four LBS motifs on the *MtRVE1* promoter that MtLUX might bind to and designed primers accordingly (Fig. 3A). The leaves of 4-week-old *MtLUXpro::MYC-MtLUX-GFP-2HA/mtlux-1* transgenic seedlings were sampled at Zeitgeber times (ZT0 and ZT12), corresponding to the start and midpoint of the light period, respectively. The results confirmed the association of MtLUX with the LBS motifs in the *MtRVE1* promoter *in vivo* (Fig. 3A). Next, we performed an electrophoretic mobility shift assay (EMSA) to test whether MtLUX directly binds to the promoter of *MtRVE1*. Based on the ChIP-qPCR results, we designed probes covering the GATWCG motif. The results confirmed that the MtLUX protein directly binds to the *MtRVE1* promoter fragment (Fig. 3B). Subsequently, a dual-luciferase reporter assay showed that the MtLUX protein directly inhibits *MtRVE1* expression (Fig. 3C and D). To validate MtLUX-mediated repression of *MtRVE1* expression, we performed qPCR to measure *MtRVE1* expression levels in the *mtlux-1* and *mtlux-2* mutants and *MtLUX-OE1* and *MtLUX-OE2* overexpression lines. We observed that *MtRVE1* expression was significantly increased in mutant lines and decreased in overexpression lines compared to the wild-type R108 (Fig. 3E). Those results indicated that MtLUX binds to the promoter of *MtRVE1* and represses its expression.

### Regulation of *MtRVE1* on auxin synthesis, root development, and nodule formation

In *A. thaliana*, the expression of the *RVE1* gene exhibits rhythmic oscillations and has been demonstrated to function as an output gene of the circadian clock. Additionally, *RVE1* serves as an integral mediator of the circadian clock and auxin signaling pathways [38]. To investigate the variant expression of the *MtRVE1* gene in *M. truncatula*, we generated transgenic seedlings harboring the *MtRVE1* promoter driving the expression of luciferase (*MtRVE1promoter::LUC*). We then sampled different detached tissues and traced the rhythmicity of luciferase activity over time. Data analysis revealed that *MtRVE1* exhibits rhythmic expression across various tissues and a phase delay occurring in the shoots (Fig. S4). To further validate these results, we sampled the shoots and roots of *Medicago* every 4 h under continuous light (LL) conditions and employed qPCR to detect the expression of *MtRVE1*. We found that the expression of *MtRVE1* in both shoots and roots displayed rhythmic oscillation, similar to *MtLUX*, with a longer oscillation period and a phase delay in the shoots compared to the roots (Fig. S4D). These revealed that *MtRVE1* is a rhythmically expressed clock gene.

**Figure 4:**
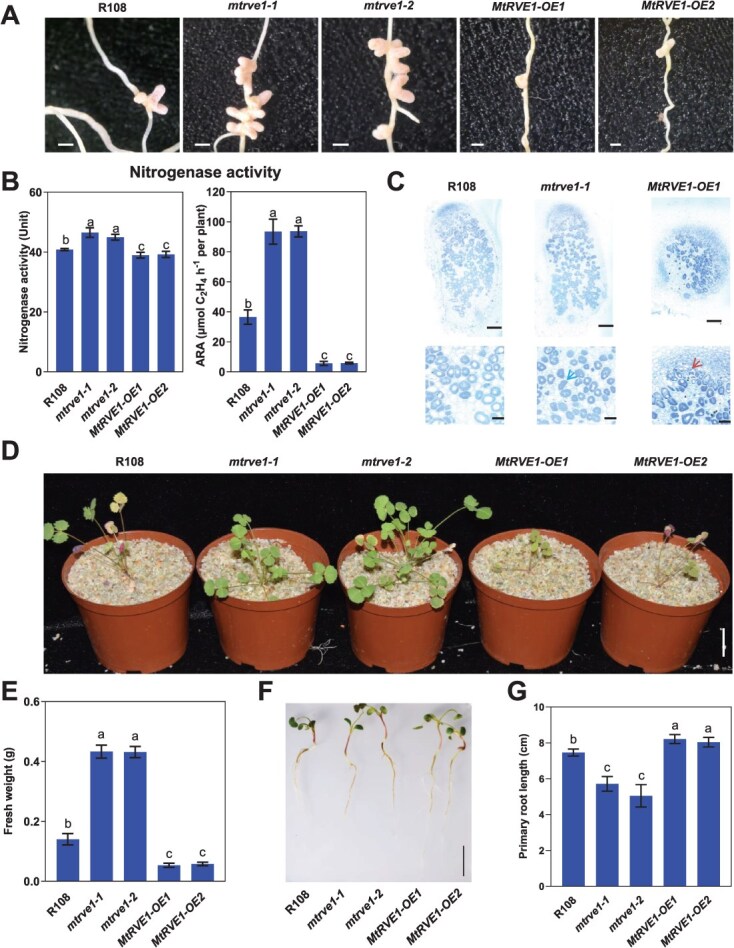
A role for *MtRVE1* in the auxin-related phenotypes of *mtrve1* mutants and *MtRVE1* overexpressing (OE) lines. (A) Growth of root nodules after 21 days of inoculation. Scale bars: 1 mm. (B) Assessment of rhizobial activity in root nodules at 21 days postinoculation. The left panel shows nitrogenase activity per nodule measured using the Plant Nitrogenase (NITS) ELISA kit. The right panel shows nitrogenase activity per plant determined by the ARA. At least 12 independent plants were analyzed. Statistical significance was determined by Student’s *t* test. Different letters indicate significant differences (*P* < 0.05). (C) Toluidine blue-stained longitudinal semithin sections of nodules from the *mtrve1* mutant and overexpression lines. Sections were prepared from resin-embedded samples and observed under light microscopy. The bottom panel shows higher-magnification views of the regions indicated in the top panel. Red arrows indicate bacteria that may be undergoing degradation. Blue arrows indicate putative bacteroids. Scale bars: 200 μm (top) and 50 μm (bottom). (D) Above-ground phenotypes of transformed lines 21 days postinoculation. Scale bars: 2 cm. (E) Analysis of fresh weight in above-ground plant parts was conducted 21 days postinoculation. Samples were collected from at least eight plant lines, and statistical analysis was performed using *t* test. Different letters ‘a–c’ indicate statistically significant differences (*P* < 0.001). (F) Root phenotypes of 10-day-old *MtRVE1* mutants and overexpressing lines. Scale bars: 1 cm. (G) Statistical analysis of primary root length. Different letters ‘a–c’ indicate significant differences in descending order. Each experiment included at least six biological replicates. Each experiment was conducted in triplicate.

Few studies have focused on *MtRVE1*, making it essential to determine its biological function through genetic approaches. We generated and characterized CRISPR-induced knockout mutants and *MtRVE1* overexpression transgenic plants. Root and nodule phenotypic analyses revealed significant changes in main root length and nodule phenotype compared to the wild type. Phenotypic analysis of nodules indicated that the *mtrve1* mutant exhibited a significantly higher nodule count compared to the wild type, whereas the *MtRVE1-OE* lines showed a markedly reduced nodule count (Fig. 4A). This suggests that *MtRVE1* may suppress nodule formation. Furthermore, nitrogenase activity assays revealed key differences relative to the R108 line, *mtrve1* mutant nodules displayed increased activity compared to the R108, while *MtRVE1*-overexpressing lines (*MtRVE1-OE*) showed significantly reduced activity (Fig. 4B). In contrast to the positive effect of *MtLUX* on nitrogenase activity, nitrogenase activity in R108 nodules was lower than in *mtrve1* mutants but higher than in *MtRVE1-OE* lines, as determined by the NITS ELISA assay (Fig. 4B, left). A similar pattern was observed for per-plant nitrogenase activity measured by the ARA (Fig. 4B, right), supporting a regulatory role of *MtRVE1* in symbiotic nitrogen fixation. Histological examination of toluidine blue-stained nodules further showed that *MtRVE1-OE* lines displayed disorganized cellular structure compared with the control (Fig. 4C).

Additionally, 21 days postinoculation, the above-ground growth of the *mtrve1* mutant was more robust than that of the wild type, whereas the *MtRVE1-OE* lines displayed weaker growth relative to R108 (Fig. 4D). Under low-nitrogen conditions, the *mtrve1* mutants exhibited significantly higher shoot biomass compared to controls, while the *MtRVE1-OE* lines displayed lower shoot biomass (Fig. 4E). This implies that *MtRVE1* could modulate biological nitrogen fixation by regulating nodule formation, which in turn affects the aerial growth of the plant.

The main root length of *MtRVE1* CRISPR/Cas9-edited knockout mutants (*mtrve1-1* and *mtrve1-2*) was significantly reduced, whereas the main root length of *MtRVE1* overexpression (*MtRVE1-OE1* and *MtRVE1-OE2*) was increased compared to the wild type (Fig. 4F and G). Furthermore, when the transgenic lines were grown in 1/2 MS medium containing 2 μM NPA, both the mutants and the overexpression lines exhibited root lengths and lateral root numbers similar to the R108 phenotype (Fig. S5), which differ from the transgenic lines growing in 1/2 MS medium (Fig. 4F). This suggests that, in contrast to the *mtlux* mutants, the *mtrve1* mutants demonstrate a phenomenon of auxin enrichment.

### *MtRVE1* functions downstream of *MtLUX* in mediating auxin-regulated root development and nodule formation.

**Figure 5:**
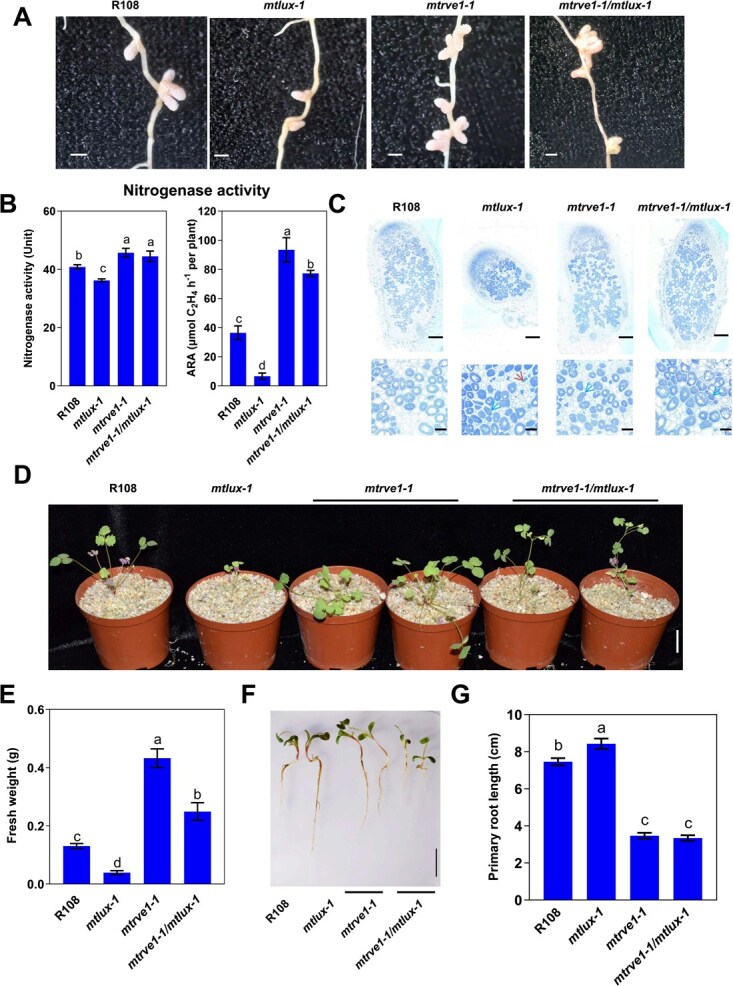
Regulation of auxin-mediated nodule formation and root growth by *MtLUX* through the inhibition of *MtRVE1* expression. (A) Growth of root nodules after 21 days of inoculation. Scale bars: 1 mm. (B) Assessment of rhizobial activity in root nodules at 21 days postinoculation. The left panel shows nitrogenase activity per nodule measured using the Plant Nitrogenase (NITS) ELISA kit. The right panel shows nitrogenase activity per plant determined by the ARA. At least 12 independent plants were analyzed. Statistical significance was determined by Student’s *t* test. Different letters indicate significant differences (*P* < 0.05). (C) Toluidine blue-stained longitudinal semithin sections of nodules from the mutant and lines. Sections were prepared from resin-embedded samples and observed under light microscopy. The bottom panel shows higher-magnification views of the regions indicated in the top panel. Red arrows indicate bacteria that may be undergoing degradation. Blue arrows indicate putative bacteroids. Scale bars: 200 μm (top) and 50 μm (bottom). (D) Above-ground phenotypes of transformed lines 21 days postinoculation. Scale bars: 2 cm. (E) Analysis of fresh weight in above-ground plant parts was conducted 21 days postinoculation. Samples were collected from at least eight plant lines, and statistical analysis was performed using *t* test. Different letters ‘a–d’ indicate statistically significant differences (*P* < 0.001). (F) Root phenotypes of 10-day-old *mtlux* and *mtrve1* mutants and overexpressing lines. Scale bars: 1 cm. (G) Statistical analysis of primary root length. Different letters ‘a–c’ indicate significant differences in descending order. Each experiment included at least six biological replicates. Each experiment was conducted in triplicate.

*MtRVE1* is a downstream effector in the *MtLUX* signaling cascade involved in auxin-mediated root development and nodule formation. To test this hypothesis, we generated and characterized double mutants with *MtRVE1* gene mutations in the *mtlux-1* mutant background. The nodule and root phenotypes of these double mutants were then investigated. The number of nodules in the *MtRVE1* CRISPR/Cas9-edited knockout mutants (*mtrve1* mutants) in both R108 and *mtlux-1* mutant backgrounds were significantly higher than that of the *mtlux-1* mutant. The nodule phenotype of the *mtrve1/mtlux-1* double mutants was more similar to that of the *mtrve1* single mutants (Fig. 5A). Furthermore, nitrogenase activity assays indicated the *mtrve1/mtlux* double mutant and *mtrve*1 single mutant both demonstrated high nitrogenase activity levels (Fig. 5B). Nitrogenase activity in *mtrve1/mtlux* nodules was higher than in R108, similar to the pattern observed in *mtrve1* (Fig. 5B, left). Per-plant activity showed the same trend (Fig. 5B, right), supporting a role for *MtRVE1* and *MtLUX* in modulating nitrogenase activity. These findings collectively indicate that *MtLUX* may regulate nitrogenase activity via modulation of *MtRVE1* expression, thereby influencing biological nitrogen fixation. Toluidine blue staining revealed partial restoration of symbiosome architecture in double mutant (*mtrve1-1/mtlux-1*) nodules, with intact structures compared with the collapsed symbiosomes in *mtlux-1* single mutants. (Fig. 5C). This structural phenotype provides additional evidence that *MtLUX* participates in nodule development through regulatory control of *MtRVE1* expression. Moreover, the *mtrve1/mtlux* double and *mtrve1* single mutants inoculated with nodule bacteria exhibited significantly better growth under low-nitrogen nutrient conditions compared to the wild type and other mutants (Fig. 5D). The *mtrve1/mtlux-1* double mutants even flowered normally under low nitrogen conditions, maintaining a normal life cycle. This indicates that the *MtRVE1* mutation enhances biological nitrogen fixation, allowing the *mtrve1/mtlux-1* double mutants to maintain the life cycle of the *mtlux-1* mutant under normal nutrient conditions. In addition, under low-nitrogen conditions, the *mtrve1/mtlux* double mutant demonstrated shoot biomass phenotypes closely resembling those of the *mtrve1* single mutant (Fig. 5E). These observations collectively suggest that *MtLUX* regulates biological nitrogen fixation in *M. truncatula* through transcriptional modulation of *MtRVE1* expression, thereby influencing shoot biomass accumulation. At the same time, bioluminescence and GUS staining analysis in plants inoculated by rhizobia confirmed high *MtLUX* expression levels, while *MtRVE1* also showed detectable expression in nodular tissues (Fig. S6). Subsequently, a schematic visualization of cell-type/tissue-specific expression was generated via the bio-analytic resource (BAR) ePlant/eFP viewer to present the spatiotemporal expression of *MtLUX* and *MtRVE1* in *M. truncatula* root tissues during symbiotic interactions with arbuscular mycorrhizal fungi and rhizobia [39]. Experimental results demonstrated that, subsequent to rhizobial inoculation, *MtLUX* and *MtRVE1* displayed significant differential regulation in their relative expression levels across distinct root regions and nodules (Fig. S7). The expression of *MtLUX* and *MtRVE1* in root nodules indicates a potential involvement in nodule development.

Similarly, the primary root length of the *mtrve1* mutants in both R108 and *mtlux-1* backgrounds was significantly shorter than that of the *mtlux-1* mutant (Fig. 5F and G). Notably, the primary root length of the *mtrve1/mtlux-1* double mutants not only reverted to the phenotype of the *mtlux-1* mutant but also more closely resembled the phenotype of the *mtrve1* single mutant, being shorter than that of the wild-type R108. This finding indicates that *MtRVE1* functions downstream of *MtLUX* in the regulation of primary root growth.

**Figure 6:**
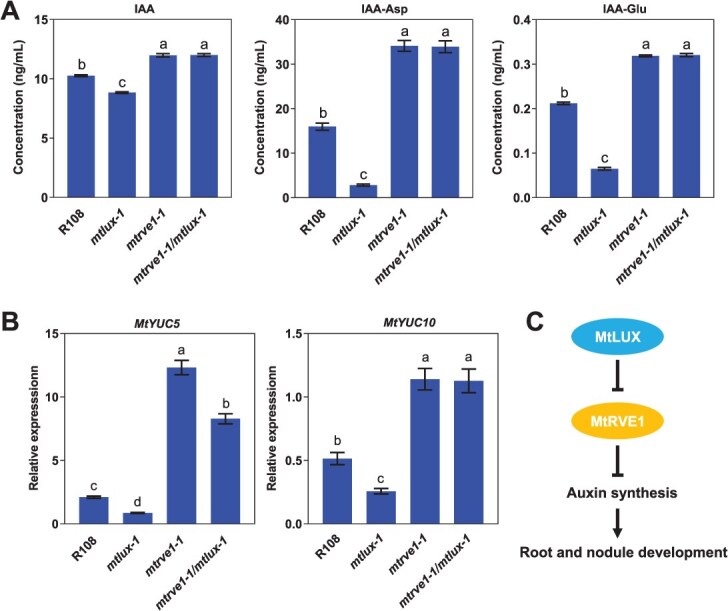
Regulation of auxin synthesis by *MtLUX* through the inhibition of *MtRVE1* expression. (A) Auxin levels in nodules of *mtlux* and *mtrve1* mutants. Using HPLC-MS/MS, we measured concentrations of free auxin IAA and two conjugated auxins (IAA-ASP, IAA-GLU) in mutant nodules. Sample collection involved ≥0.5 g per group with two or more biological replicates. Statistical analysis employed *t* test, with different letters ‘a–c’ denoting significant differences (*P* < 0.001). (B) Expression analysis of auxin biosynthesis genes *MtYUC5* (A) and *MtYUC10* in roots. The relative transcript levels were normalized to the abundance of the reference gene *MtACTIN* and are presented as means ± SD of three biological replicates (*n* = 3). Different letters ‘a–d’ indicate significant differences in descending order. Each experiment was conducted in triplicate. (C) MtLUX binds to the *MtRVE1* promoter to regulate auxin synthesis in *M. truncatula*, resulting in increased primary root and nodule development.

Additionally, previous experiments indicated the *mtlux* mutants exhibited an auxin-deficient phenotype, while the *mtrve1* lines displayed an auxin accumulation phenotype. Using HPLC-MS/MS, we quantified free IAA and conjugated auxins (IAA-Glu/IAA-Asp) in root nodules. The results revealed that *mtlux* mutant nodules exhibited significant reductions in both free and conjugated auxin levels, whereas *mtrve1* single mutant and *mtlux/mtrve1* double mutant displayed exclusive elevation in conjugated auxins (IAA-Glu/IAA-Asp) and free IAA concentrations (Fig. 6A). These findings suggest that *MtLUX* may modulate the biosynthesis of specific auxin conjugates via regulation of *MtRVE1*, thereby participating in the regulatory network of nodule formation and development. Subsequent analysis of auxin biosynthesis genes in the double mutants revealed that the expression of *YUC* genes (*YUC5* and *YUC10*) in the *mtrve1/mtlux-1* double mutants was similar to that in *mtrve1/R108*, both showing a significant increase (Fig. 6B). *MtRVE1* may participate in plant growth and development by inhibiting auxin biosynthesis. In conclusion, the inhibition of *MtRVE1* expression by the MtLUX protein connects the circadian clock with auxin, proposing a new mechanism for the circadian regulation of nodules in *Medicago*: the *MtLUX*-*MtRVE1*-*MtYUCs* pathway (Fig. 6C).

## Discussion

In mammals, research underscores the gut microbiome’s crucial role in maintaining circadian rhythm stability in the gut and synchronizing with the brain’s clock, especially in response to shifts in day–night cycles [40]. Conversely, disruptions within the host’s circadian system can lead to changes in gut microbiota communities, which in turn can disrupt metabolic processes, energy homeostasis, and provoke inflammatory responses [41, 42]. These findings naturally inspire botanists to explore whether nitrogen-fixing rhizobia are similar to gut microbes, with their biological rhythms regulating the host’s biological rhythms. Conversely, it is worth investigating whether the plant’s biological rhythms affect the formation of root nodules, the rhythms of nitrogen-fixing bacteria, and the regulation of biological nitrogen fixation [43, 44]. Because most research on plant circadian clocks has primarily utilized the traditional model plant *Arabidopsis*, and studies on biological rhythms in leguminous plants have only recently been initiated, the question of whether plant circadian clocks regulate the formation of rootnodules and nitrogen fixation has long been an unresolved and highly scrutinized issue within the field of chronobiology. Despite recent studies indicating that the circadian clock’s core oscillator genes regulate nodule formation, the underlying mechanisms remain largely unknown. Additionally, the circadian clock likely regulates nodule formation through various mechanisms at multiple levels. This study identifies the key gene *MtRVE1*, which governs root development and nodule formation, using a combination of biochemical and genetic approaches. Notably, this research is the first to investigate *MtRVE1* in legumes, demonstrating its role in nodule formation. Although previous studies have explored the circadian clock’s regulation of hormone synthesis, its biological significance has not been fully elucidated, and its influence on nodulation through auxin biosynthesis has not been reported until now. This paper identifies *MtRVE1* as a crucial link between auxin biosynthesis and the circadian clock, suggesting it regulates auxin levels by inhibiting the expression of auxin synthesis genes. Furthermore, the inhibition of *MtRVE1* expression by the *MtLUX* protein connects the circadian clock with auxin, proposing a new mechanism for the circadian regulation of nodules in *Medicago*: the *MtLUX*-*MtRVE1*-*MtYUC*s pathway (Fig. 6C).

The circadian clock, by regulating the synthesis and action of hormones, aids plants in adapting to periodic environmental changes such as light–dark cycles and temperature fluctuations. This regulatory function is instrumental in enabling plants to grow and develop at the appropriate times, thereby enhancing their adaptability and survival capabilities [45, 46]. In fact, the synthesis and metabolism of most hormones are governed by the circadian clock [47]. This implies that at different times of the day, the concentrations of various hormones within plants exhibit cyclical variations, which in turn influence the physiological and metabolic processes of the plants. For instance, during the daytime, when auxin levels peak, hormones are not only regulated by the circadian clock but also provide feedback to modulate the clock. Hormones may affect the expression or activity of core components of the circadian clock, thereby impacting its rhythmicity. The interplay between plant hormones and the circadian clock constitutes a complex regulatory network that governs plant growth and development as well as environmental adaptation. By delving deeper into these interactions, we can gain a better understanding of the physiological mechanisms in plants and provide a theoretical foundation for agricultural production and crop improvement.

Root morphology, which has been comparatively less studied, is likely regulated by the circadian clock through its influence on auxin distribution and the roots’ gravitropic and hydrotropic responses. Root morphology is crucial for the plant’s water and nutrient uptake, suggesting that circadian modulation of root development may indirectly affect the overall growth and developmental processes of the plant. The circadian clock may regulate nitrogen fixation in plants in a tissue-specific manner. *MtRVE1* is predominantly expressed in roots and shoots (Fig. S4), while *LUX* is expressed in both above-ground and below-ground tissues, indicating that the circadian clock’s regulation of biological activities in different tissues and organs may be tissue specific. The enzymes encoded by the *YUC* genes are pivotal in the biosynthetic pathway of auxin. Extensive research has conclusively shown that various members of the *YUC* genes family possess the capacity to catalyze the conversion of tryptophan into auxin, specifically indole-3-acetic acid (IAA), which is the predominant form of auxin in plants [48–50]. In woodland strawberry (*Fragaria vesca*), *FveYUC4* is crucial for morphogenesis in both leaves and flowers of by ensuring the timely and tissue-specific provision of auxin hormone [51]. In wheat, seed-specific expression of *TaYUC10* results in a significant increase in auxin and protein content within wheat seeds [52]. In cucumber, *CsYUC10b* reduced auxin accumulation, leading to spatial variation [53]. Various model plants have exhibited distinct expression patterns of *YUC* genes, which function in the regulation of auxin synthesis. Despite the limited research conducted on *YUC* genes in *M. truncatula*, insights derived from other model organisms provide a plausible basis for hypothesizing that the *MtYUC* genes may also be involved in the regulation of auxin synthesis. Recent studies have demonstrated that auxin plays a pivotal role in influencing root nodule formation, exhibiting species-specific mechanisms among various legume species [14, 16–18]. Considering this, *MtRVE1* possibly negatively regulates *MtYUC5/10*, which in turn modulates auxin synthesis, ultimately leading to decreased nodule formation. However, conducting thorough research in this area necessitates substantial time and financial investments for in-depth exploration.

The regulatory function of the circadian clock is multilevel and multidimensional. It ensures that life activities can operate and maintain normalcy under various complex environmental conditions through a complex network of interconnections and feedback mechanisms. Therefore, we can reasonably speculate that the circadian clock can regulate nodulation and nitrogen fixation in leguminous plants through various pathways. Auxin, along with other endogenous plant hormones such as cytokinin and gibberellins, plays a pivotal role in modulating the initiation and progression of nodule formation. The circadian clock, a fundamental regulatory entity, exerts control over the synthesis and signal transduction pathways of numerous hormones. It is observed that the concentrations of several hormones exhibit temporal peaks at distinct daily intervals. The mechanism by which the circadian clock orchestrates the synergistic actions of these hormones to modulate nodule development is a topic of significant scientific interest. Furthermore, this research endeavor offers crucial theoretical underpinning for the exploration and development of novel nitrogen-fixing varieties of the model legume plant, *M. truncatula*, within the framework of circadian rhythm studies.

We propose a model in which *LUX/RVE1* may influence nodulation onset by coordinating circadian rhythms with auxin dynamics (Figs 6A, B, S1A, and  S4A). Current evidence indicates that this effect likely involves the interplay between circadian regulation and auxin signaling pathways rather than direct targeting of nodulation-specific genes. Nitrogenase activity assays, including ELISA and ARA measurements, support a role for *LUX/RVE1* in modulating root nodule function, providing valuable phenotypic insights despite the absence of identified direct molecular targets. The use of spatiotemporal transcriptomics and functional genetics offers a framework for dissecting cell-type-specific regulatory mechanisms. Future studies employing tissue-specific complementation and chromatin interaction analyses will be necessary to validate this framework and pinpoint direct targets. Although direct molecular interactions remain to be determined, our results highlight the potential of *LUX/RVE1* as an integrator of endogenous rhythmic signals and provide a foundation for understanding how circadian mechanisms shape symbiotic processes, ultimately informing strategies to optimize crop symbiotic efficiency.

## Materials and methods

### Plant materials and growth conditions

The *M. truncatula* line, R108 and CRISPR/Cas9-edited mutant *mtlux-1*, served as parent lines to generate overexpression lines and CRISPR/Cas9-edited mutants. After being scarified with sandpaper, the seeds were sterilized with 1% sodium hypochlorite for 30 min. After being washed with ddH_2_O four to five times, the seeds were placed on/transferred to 1/2 MS media (pH 5.7–5.9) containing 1% sucrose and 1% agar for 7 days in the dark at 4°C. Seedlings were grown in a light incubator, under a 12-h light/12-h dark photoperiod at a constant temperature of 22°C, and relative humidity of 60%–80% for 10 days, and then transferred to a long-day (16-h light/8-h dark) greenhouse.

### Construction of complementary transformed plants and RNA-seq data analysis

We cloned 2461-bp genomic sequences upstream of the CDS starting site and inserted them into the pCAMBIA-1300 vector. *mtlux*-complementary transformed plants were obtained by tissue culture transformation. We collected the roots of 4-week-old seedlings at ZT12h to do RNA-seq. GraphPad Prism 8 was used to analyze qPCR data. KEGG, GO, and WikiPathways [54] were analyzed by the online software Metascape (http://metascape.org/gp/index.html#/main/step1) [55].

### Vectors construction and *Medicago truncatula* transformation

In order to construct overexpression vectors, *MtLUX* or *MtRVE1* CDS sequences were cloned and ligated into the PMDC32 vector. For generating CRISPR/Cas9 constructs, the genomic DNA target fragments linking (gRNA-Sc)-(U6-26t)-(MtU6-6) were amplified from the DT1T2 by PCR and cloned into the pMtCRISPR-GFP vector [36]. The target sites were selected using the online tool CRISPR2 (http://crispr.hzau.edu.cn/CRISPR2/) [56]. To construct the *MtRVE1pro::GUS* vector, the *MtRVE1* promoter was amplified from R108 genomic DNA by PCR and inserted into the pCAMBIA3301 vector. The promoter, firefly luciferase gene, and CaMV term were cloned and lined into pCAMBIA1300. *Agrobacterium*-mediated transformation was performed using the leaves of 4-week-old plants; detailed methods were performed in accordance with the protocol [57]. The primers are listed in Table S1.

### RNA extraction and qRT-PCR

Total RNA was extracted from 10-day-old Medicago seedlings using the MagicPure® Total RNA Kit (TransGen, Beijing, China). Five hundred nanograms of total RNA was used for All-in-One First-Strand Synthesis MasterMix (with dsDNase) (Guangzhou Xinkailai Biotechnology Co. Ltd, Guangzhou, China). Quantitative real-time PCR analysis was performed using TransScript® II Probe One-Step qRT-PCR SuperMix (TransGen, Beijing, China) in a CFX Connect Real-Time System (Bio-Rad, Singapore) following the manufacturer’s instructions. *MtActin* was used as an internal control. The qRT-PCR assays were performed with three biological replicates, and three technical replicates were performed in each biological replicate. Information regarding the primers used in this assay are available in Table S1.

### Dual-luciferase transient expression assays

The full-length *MtLUX* CDS was cloned into the pGreen II 62-SK vector to generate the effector vectors, which were driven by the cauliflower mosaic virus 35S promoter. The ~2 kb *MtRVE1* promoter sequence was cloned into the pGreen II 0800 vector driving firefly luciferase to generate the reporter vector. *Renilla* luciferase driven by a full-length cauliflower mosaic virus 35S (CaMV35S) promoter served as an internal control. Vectors were transformed into epidermal cells of *Nicotiana benthamiana* leaves for transient expression as described previously [58]. The firefly luciferase and *Renilla* luciferase activities were measured using the Berthold TriStar^2^ S LB 942 multimode reader (BERTHOLD TECHNOLOGIES, Wildbad, Germany) according to the instruction manual of Dual Luciferase Reporter Gene Assay Kit (Guangzhou Xinkailai Biotechnology Co. Ltd, Guangzhou, China).

### Chromatin immunoprecipitation assay

Leaf samples were harvested from 3-week-old transgenic plants at zeitgeber time (ZT) 0h and ZT12h. ChIP-qPCR assays using *MtLUXpro::MYC-MtLUX/mtlux-1* lines were performed as previously described [59]. An anti-MYC antibody (Sigma-Aldrich, M4439) was used for the ChIP assay after formaldehyde-mediated crosslinking, chromatin isolation, sonication, and immunoprecipitation. The data were analyzed by the Comparative Delta–delta Ct method. The primers are listed in Table S1.

### Electrophoretic mobility shift assay

The full-length CDS of *MtLUX* was cloned into the pGEX-4 T-1 vector. The GST-fused *MtLUX* plasmids were transformed into *Escherichia coli* BL21 (DE3) cells. Protein purification was performed as previously described [60, 61]. Probes containing the LBS were labeled with biotin at the 3′ end. The MtLUX binding site was attttatgtcttgaattctcagctactGATTCGctattgttccaatgaatcacacaaaa, and the mutant site was attttatgtcttgaattctcagctactAGCCTActattgttccaatgaatcacacaaaa. According to the manufacturer’s instructions, the EMSA assay was conducted with Light Shift Chemiluminescent EMSA kit (Thermo Fisher Scientific, 89 880).

### Genetic crossing of *Medicago truncatula*

In the hybridization process of *M. truncatula*, the initial step consists of identifying the maternal plant *DR5::VENUS*/*R108* and selecting flowers at an optimal growth stage for meticulous manual emasculation under a microscope. This procedure necessitates the complete removal of pollen from the maternal florets, which are in an immature stage, thereby allowing the simultaneous removal of anthers. Subsequently, florets from the paternal barrelclover *mtlux-1*, distinguished by their still-closed keel petals, are chosen for pollen collection. The collected pollen is then precisely utilized to pollinate the emasculated maternal florets under a microscope. Upon the completion of pollination, labels are attached to the pollinated plants, and comprehensive records are maintained to accurately document the entire process [62].

### Inoculation with rhizobia

Seeds were sown in accordance with established protocols for the cultivation of *M. truncatula*. Three-day-old seedlings were subsequently transferred to a substrate consisting of 2- to 3-mm quartz sand. Inoculation with the rhizobial strain *Sinorhizobium meliloti* 2011 was conducted once cotyledons had fully expanded and the first true leaf had emerged. The inoculation procedure followed methodologies outlined in the literature [63]. Twenty-one days postinoculation, the growth status of both the plants and the rhizobia was evaluated to assess the effectiveness of the inoculation.

### GUS staining

We cloned genomic sequences upstream of the *MtRVE1* CDS starting site and inserted them into the pCAMBIA-3301 vector. *MtRVE1pro::GUS* transformed plants were obtained by tissue culture transformation. Twenty-one days postinoculation seedlings, along with leaves, flowers, and nodules, were subjected to GUS staining. Samples were treated with 80% (v/v) acetone at −20°C for 1 h to achieve preliminary fixation. The acetone was removed, and samples were washed three times with GUS washing buffer (50 mM/l NaH_2_PO_4_·H_2_O, 50 mM/L Na_2_HPO_4_·H_2_O, 0.5 mM/L K_3_[Fe (CN)_6_], 0.5 mM/L K_4_[Fe (CN)_6_], 10 mM/L EDTA, 0.1% TritonX-100) on a rocking platform at 150 rpm for 5 min per wash to ensure complete removal of residual acetone. Following buffer removal, samples were incubated overnight at 37°C in GUS working buffer (1 mg x-gluc in 1 ml GUS washing buffer) to facilitate enzymatic reaction. After incubation, samples were treated with 70% ethanol for 2–3 h for destaining, followed by microscopic imaging of the stained tissues. After the staining procedure, the wash solution was removed, and the stained samples were prepared for imaging using both a camera and a microscope. The primers are listed at Table S1.

### Measurement of nitrogenase activity

Nitrogenase activity was estimated by Plant Nitrogenase (NITS) ELISA Kit and acetylene reduction activity (ARA). Ten root nodules were lysed in 500 μl of plant cell lysis buffer (50 mM Tris–HCl, pH 8.0, 150 mM NaCl, 1% Triton X-100, 0.5% sodium deoxycholate, 0.1% SDS, and 1 mM EDTA). Samples were incubated on ice for 5 min to ensure complete cell lysis. The lysates were then centrifuged at 10000–16000 rpm for 1 min, and the supernatant was collected. Nitrogenase activity was measured following the protocol of the Plant Nitrogenase (NITS) ELISA Kit (mlbio, Cat# ML-E-42892). Enzyme activity was quantified using a standardized ELISA protocol. Gradient concentration standards (12.5–200 U/L) were prepared by serial dilution (16×, 8×, 4×, 2×) or direct use of undiluted standard. Sample processing employed a 5-fold dilution protocol (40 μl diluent +10 μl sample) with careful pipetting to avoid bubble formation. The assay utilized a two-step incubation program (37°C, 30 min each) for antigen–antibody binding and signal enhancement, followed by a five-step washing protocol using 30× diluted wash buffer with 30 s soak cycles to achieve background absorbance ≤0.1. Chromogen addition followed a sequential protocol (50 μl chromogen A then B) with 15 min dark incubation at 37°C. Absorbance measurements at 450 nm were completed within 15 min posttermination. Enzyme activity was calculated via standard curve interpolation and normalized using lysis buffer volume and dilution factors to determine per-nodule activity.

Fresh roots bearing attached nodules were collected from four plants and transferred into 20 ml glass vials sealed with rubber septa for measuring ARA. Acetylene (2 ml) was injected into each vial, and the vials were incubated at 28°C for 1 h. Finally, a 100 μl gas sample was analyzed to quantify ethylene production via gas chromatography using a GC6890N instrument (Agilent Technologies, Les Ulis, France). For each experimental condition, at least 16 plants were analyzed [64, 65].

## Supplementary Material

Web_Material_uhag124

## Data Availability

The raw sequence data of RNA-seq reported in this paper have been deposited in the Genome Sequence Archive (PRJCA037385) in National Genomics Data Center (https://ngdc.cncb.ac.cn/gsa).
