# Supplementary material for: Role of the MtLUX-MtRVE1 regulatory module in auxin-mediated root development and nodule formation in Medicago truncatula
Source: Hortic Res. 2026 Apr 6;13(8):uhag124. doi: 10.1093/hr/uhag124 (PMC13401420; doi:10.1093/hr/uhag124)
Supplement: Web_Material_uhag124 [file web_material_uhag124.zip › Supplementary Figures.pdf]

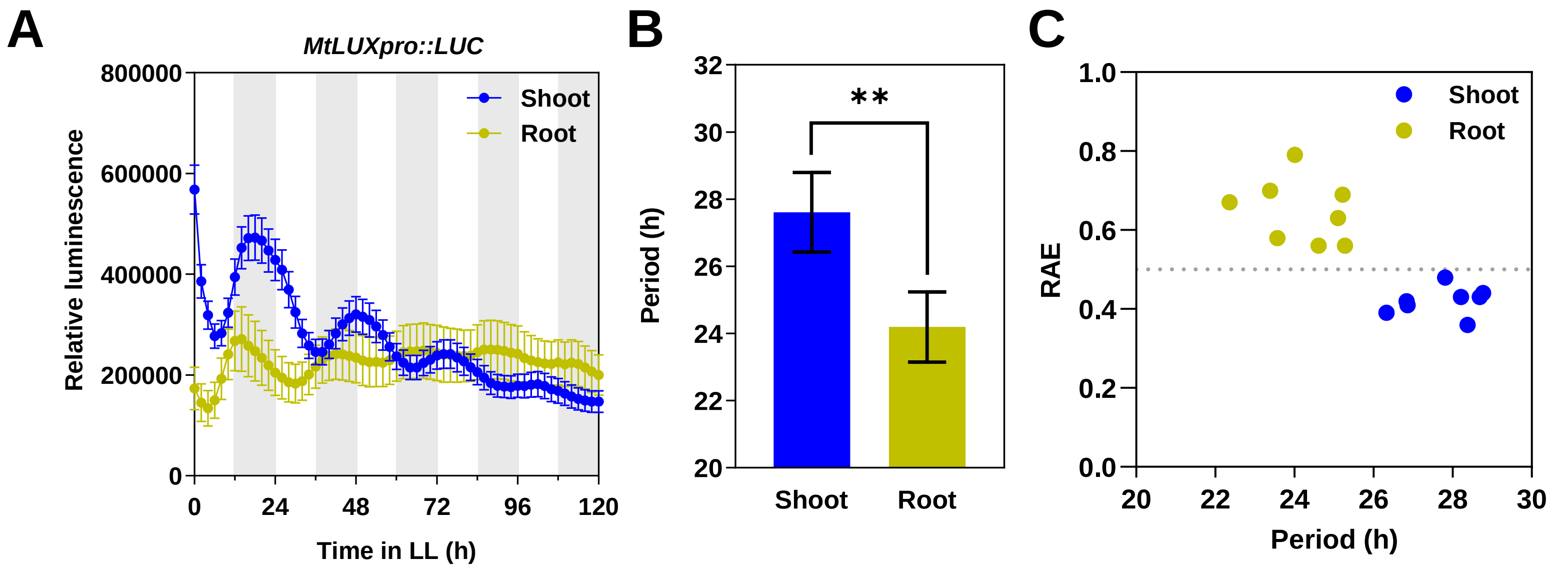

Figure S1. A role for *MtLUX* in the circadian. (A) and (B) Rhythmic changes in *MtLUX* expression in various tissues were investigated through *MtLUXpromoter::LUC* transgenic fluorescence detection. (A) Represents the rhythmic change curve, with light gray indicating the dark period under continuous light conditions. (B) Oscillation period of fluorescence detection in different tissues. (C) The error value associated with the oscillation period.

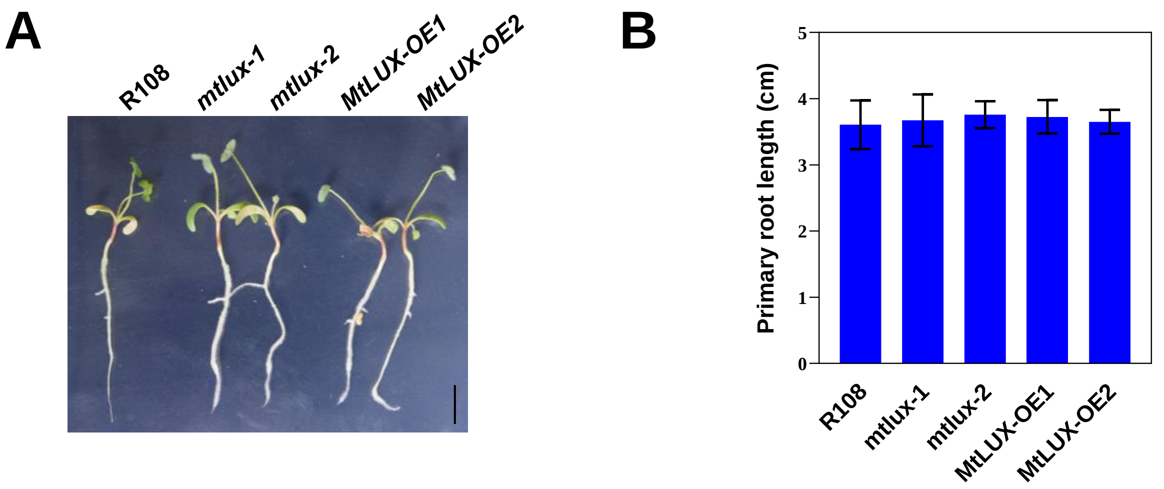

Figure S2. Root phenotypes of *MtLUX* transformed 10-day-old seedlings treated with 2 μM NPA. (A) 3-day-old seedlings were grown on 1/2 MS medium with 2 μM NPA. (B) Data were shown as mean ± SE, n>8.

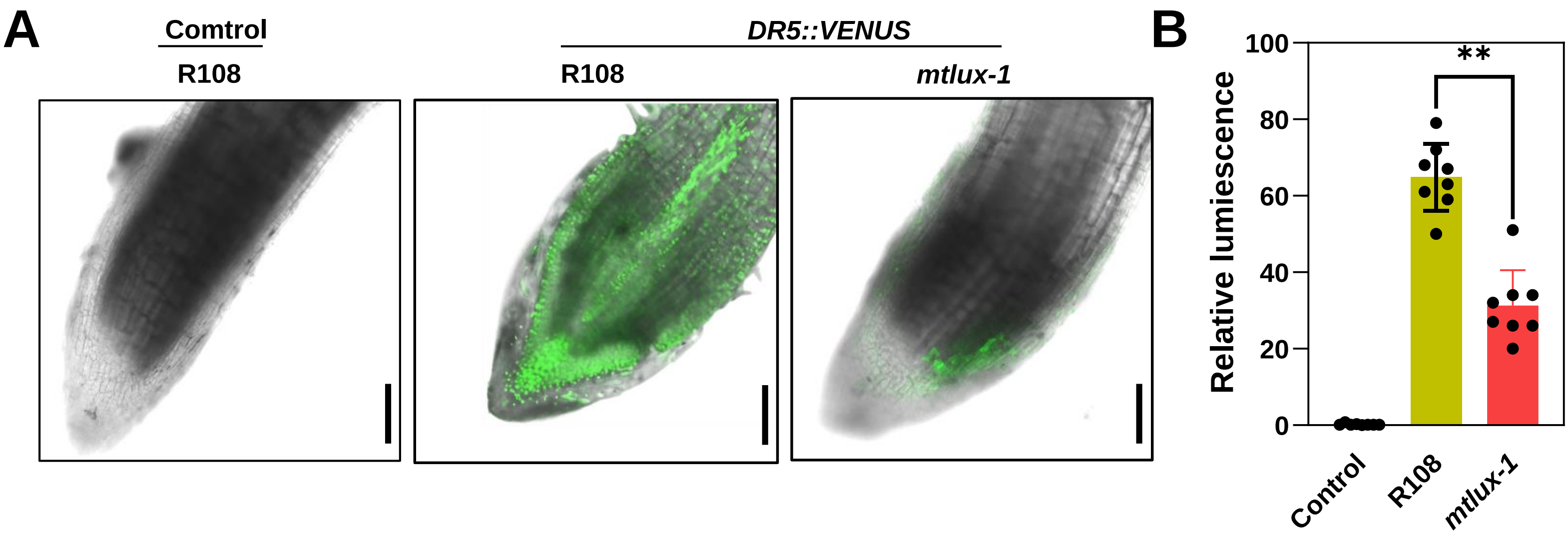

Figure S3. Analysis of fluorescence intensity in the primary root tips of wild-type R108 and *mtlux-1* mutant plants. (A) Venus fluorescence intensity in the primary root tips of *DR5::VENUS*/*R10*8 transgenic lines and hybrid seedlings derived from *DR5::VENUS*/*R108* and *mtlux*-*1* lines. Scale bars: 150 µm. (B) Analysis of the fluorescence intensity of *DR5::VENUS*. All data were obtained from at least eight biological replicates, and each experiment was conducted in triplicate.

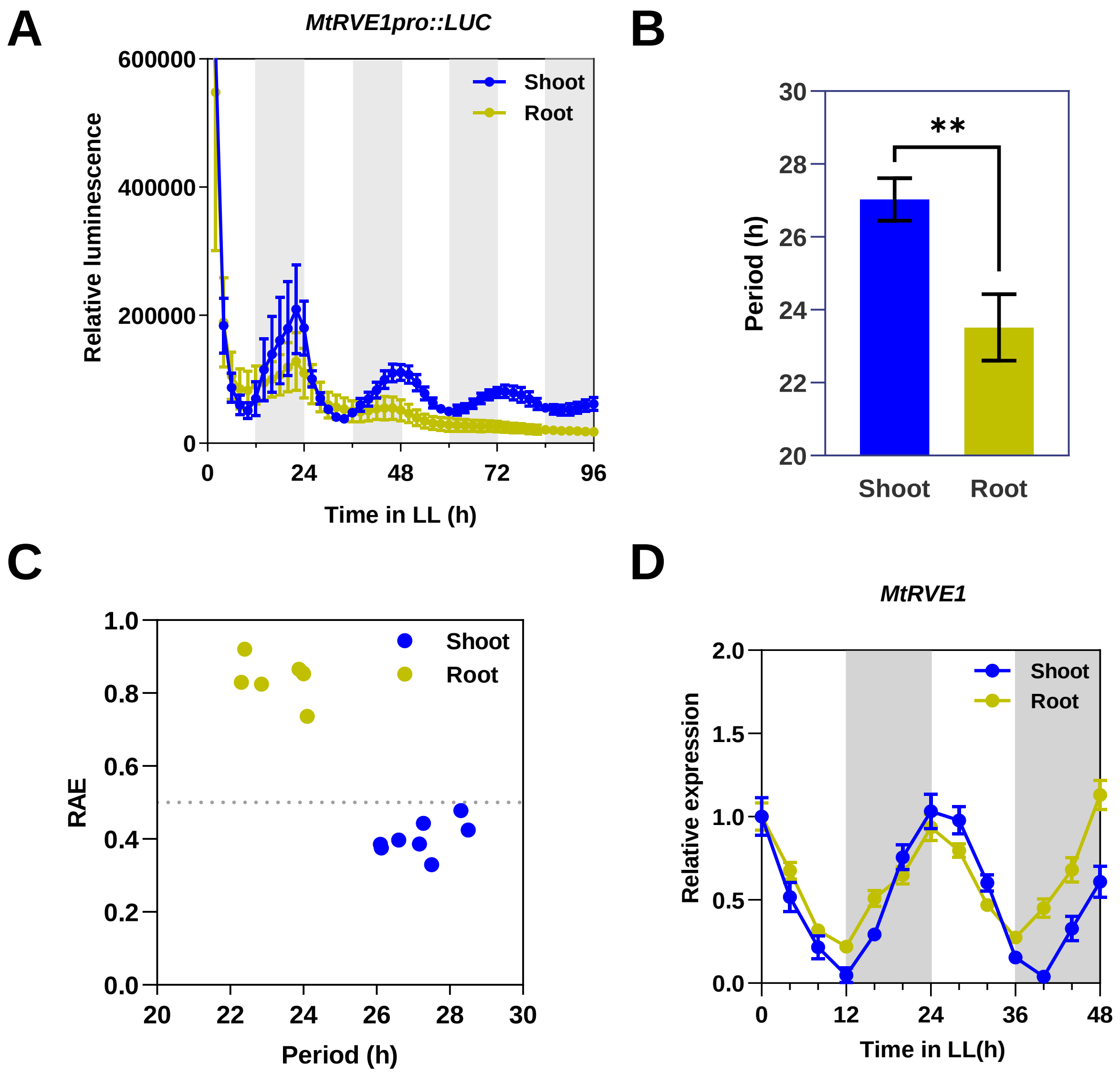

Figure S4. A role for *MtRVE1* in the circadian. (A) *MtRVE1promoter::LUC* luciferase activity in leaf and root under continuous light (LL) conditions. (B) Oscillation period detected by fluorescence in the leaf and root. (C) Error values of the oscillation period detected by fluorescence. Ten-day-old seedlings were used for the study. (D) The expression levels of *MtRVE1* in shoots and roots of R108 under LL conditions. Gene expression was measured using RT-qPCR. Relative transcript levels were normalized to *MtACTIN* and presented as means ± SD from three biological replicates (n=3), and each experiment was conducted in triplicate. Dark periods are indicated in light gray under continuous light conditions.

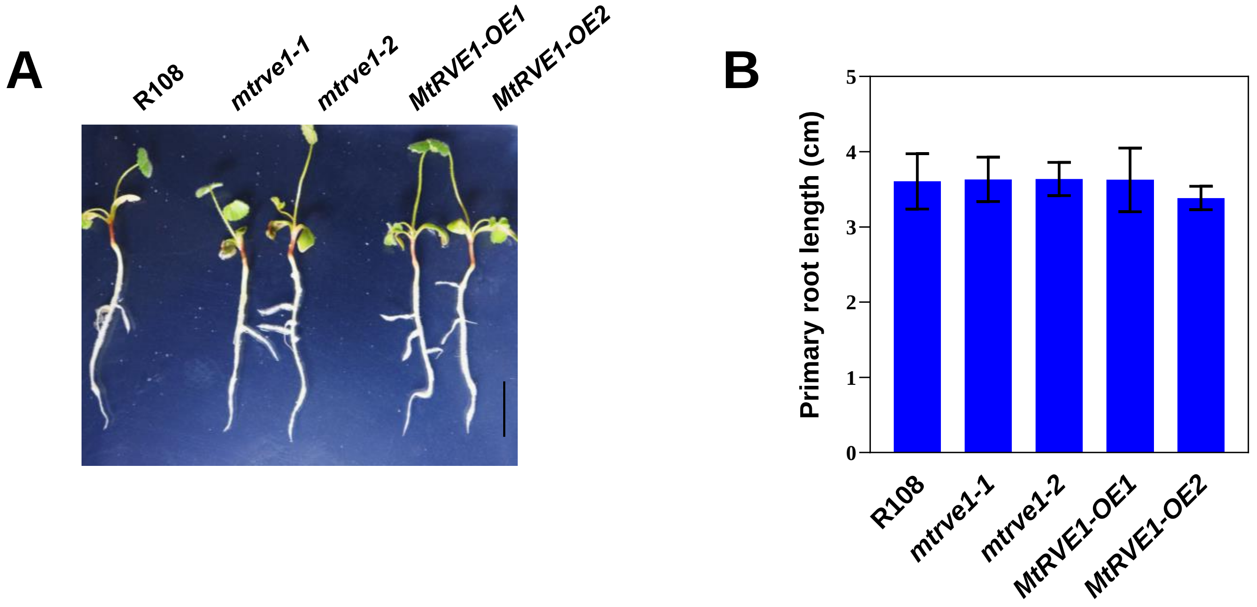

Figure S5. Root phenotypes of *MtRVE1* transformed 10-day-old seedlings treated with 2 μM NPA. (A) 3-day-old seedlings were grown on 1/2 MS medium supplemented with 2 μM NPA. (B) Data are presented as mean ± SE, n > 8. Each experiment was conducted in triplicate.

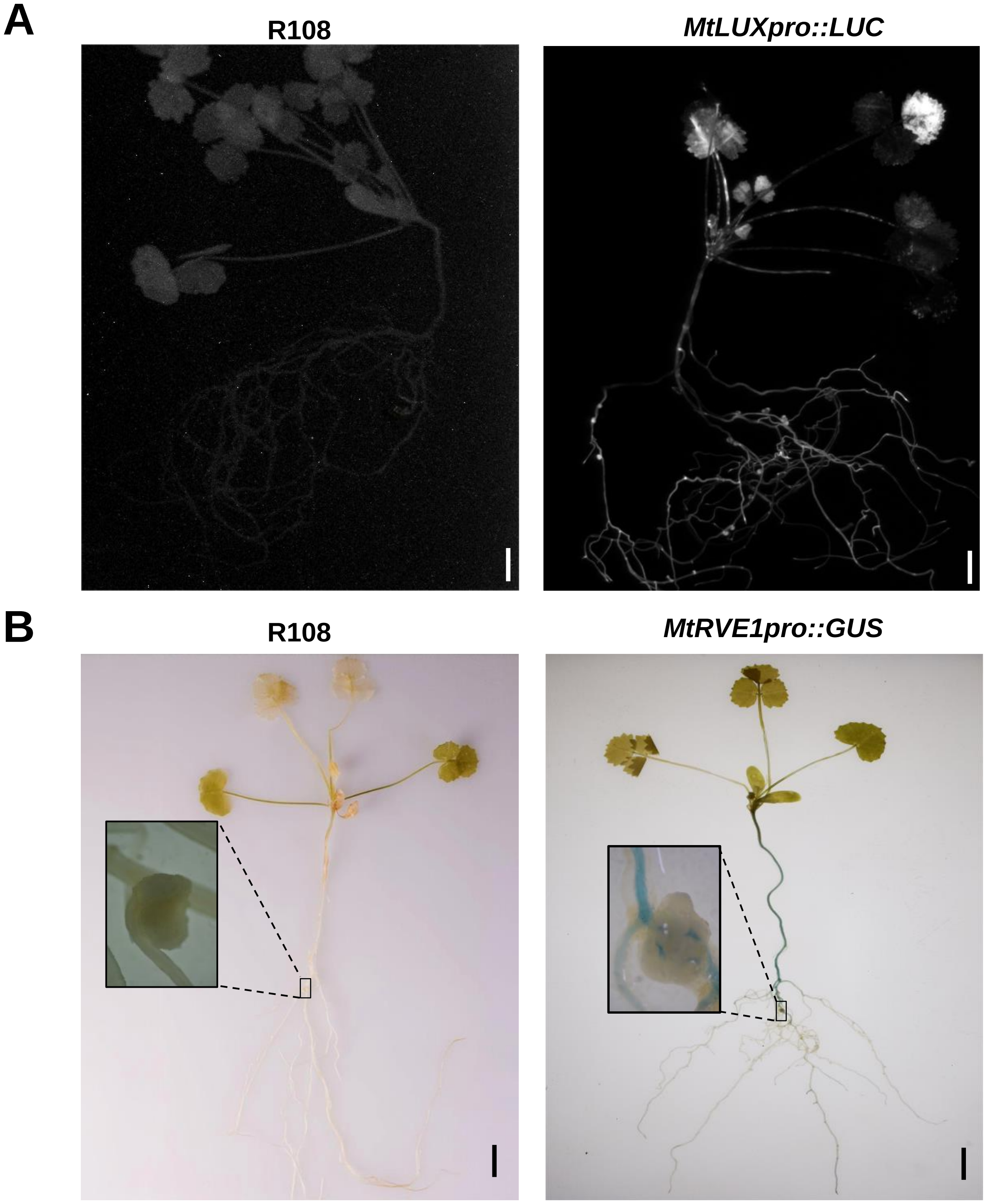

Figure S6. Expression analysis of *MtLUX* and *MtRVE1* in nodules. Plant materials inoculated with rhizobia for 21 days were used for analysis. (A) Live imaging of *MtLUXpro::LUC* transgenic lines using a bioluminescence imaging system. Results demonstrate that under low-nitrogen conditions, *LUX* is expressed in all organs, with particularly strong expression in nodules and leaves. (B) GUS staining of *MtRVE1pro::GUS* transgenic lines. Results show that under low-nitrogen or nitrogen-free conditions, *MtRVE1* is expressed in all organs, with prominent expression in roots and stems, and moderate expression in nodules. Labeled area is 5×magnification. Scale bars: 1 cm.

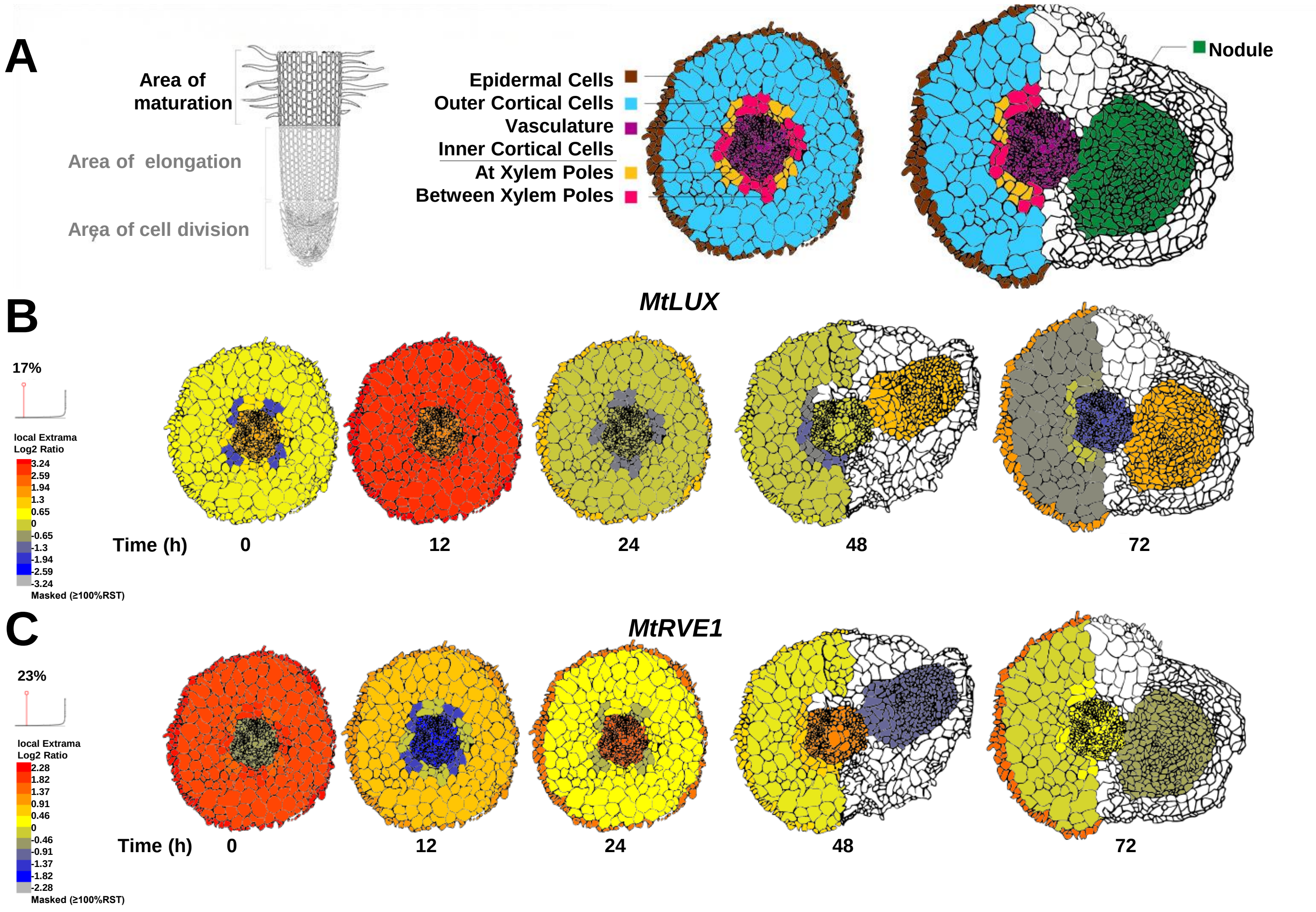

Figure S7. Spatiotemporal expression profiling of MtLUX and MtRVE1 in Medicago truncatula root tissues during symbiotic interactions with arbuscular mycorrhizal fungi and rhizobia. (A) Schema for displaying tissue expression employing default settings at https://bar.utoronto.ca/eplant_medicago/. Data are compiled from three independent replicates and colors are painted on an entire tissue regardless of which cells within that tissue expressed the gene. (B) and (C) Relative expression levels of MtLUX and MtRVE1 across distinct root tissues at specified time points post-inoculation.
